# Longitudinal COVID-19 Surveillance and Characterization in the Workplace with Public Health and Diagnostic Endpoints

**DOI:** 10.1128/mSphere.00542-21

**Published:** 2021-07-07

**Authors:** Manjula Gunawardana, Jessica Breslin, John M. Cortez, Sofia Rivera, Simon Webster, F. Javier Ibarrondo, Otto O. Yang, Richard B. Pyles, Christina M. Ramirez, Amy P. Adler, Peter A. Anton, Marc M. Baum

**Affiliations:** a Department of Chemistry, Oak Crest Institute of Science, Monrovia, California, USA; b University of California, Los Angeles, Division of Infectious Diseases, Department of Medicine, David Geffen School of Medicine at UCLA, Los Angeles, California, USA; c University of California, Los Angeles, Department of Microbiology, Immunology, and Molecular Genetics, David Geffen School of Medicine at UCLA, Los Angeles, California, USA; d Department of Pediatrics, University of Texas Medical Branch, Galveston, Texas, USA; e Department of Microbiology and Immunology, University of Texas Medical Branch, Galveston, Texas, USA; f University of California, Los Angeles, Department of Biostatistics, UCLA Fielding School of Public Health, Los Angeles, California, USA; g Jumpstart Research Consulting, LLC, Santa Fe, New Mexico, USA; University of Maryland School of Medicine

**Keywords:** SARS-CoV-2, antibodies, asymptomatic, long-term infection, serology, surveillance, workplace

## Abstract

Public health practices and high vaccination rates currently represent the primary interventions for managing the spread of coronavirus disease 2019 (COVID-19). We initiated a clinical study based on frequent, longitudinal workplace disease surveillance to control severe acute respiratory syndrome coronavirus 2 (SARS-CoV-2) transmission among employees and their household members. We hypothesized that the study would reduce the economic burden and loss of productivity of both individuals and small businesses resulting from standard isolation methods, while providing new insights into virus-host dynamics. Study participants (27 employees and 27 household members) consented to provide frequent nasal or oral swab samples that were analyzed by reverse transcription-quantitative PCR (RT-qPCR) for SARS-CoV-2 RNA. Two study participants were found to be infected by SARS-CoV-2 during the study. One subject, a household member, was SARS-CoV-2 RNA positive for at least 71 days and had quantifiable serum virus-specific antibody concentrations for over 1 year. One unrelated employee became positive for SARS-CoV-2 RNA over the course of the study but remained asymptomatic, with low associated viral RNA copy numbers, no detectable serum IgM and IgG concentrations, and IgA concentrations that decayed rapidly (half-life: 1.3 days). A COVID-19 infection model was used to predict that without surveillance intervention, up to 7 employees (95% confidence interval [CI] = 3 to 10) would have become infected, with at most 1 of them requiring hospitalization. Our scalable and transferable surveillance plan met its primary objectives and represents a powerful example of an innovative public health initiative dovetailed with scientific discovery.

**IMPORTANCE** The rapid spread of SARS-CoV-2 and the associated COVID-19 has precipitated a global pandemic heavily challenging our social behavior, economy, and health care infrastructure. In the absence of widespread, worldwide access to safe and effective vaccines and therapeutics, public health measures represent a key intervention for curbing the devastating impacts from the pandemic. We are conducting an ongoing clinical study based on frequent, longitudinal workplace disease surveillance to control SARS-CoV-2 transmission among employees and their household members. Our study was successful in surveying the viral and immune response dynamics in two participants with unusual infections: one remained positive for SARS-CoV-2 for 71 days, while the other was asymptomatic, with low associated viral RNA copy numbers. A COVID-19 infection model was used to predict that without surveillance intervention, up to 7 employees would have become infected, with at most 1 of them requiring hospitalization, underscoring the importance of our program.

## INTRODUCTION

The rapid spread of severe acute respiratory syndrome coronavirus 2 (SARS-CoV-2) and the associated coronavirus disease 2019 (COVID-19) has precipitated a global pandemic heavily challenging our social behavior, economy, and health care infrastructure.

Worldwide, recovery from this active, devastating outbreak cannot begin until safe and effective medicines for treatment and prevention are available and accessible globally. In the interim, it is essential to use the rapidly expanding scientific knowledge on the novel SARS-CoV-2 to update guidelines for COVID-19 patient management as well as protection of the uninfected population. The implementation of social distancing, frequent and thorough hand washing, isolation, and the use of face coverings (1) has helped curb the incidence of COVID-19 in many parts of the United States, and these measures continue to be important until high vaccination rates have been achieved. Our ability to design and introduce effective public health measures to reduce the spread of COVID-19 relies on an understanding of the viral pathogenesis and dynamics, fundamental aspects of the disease that remain largely unknown.

Workplace SARS-CoV-2 transmission is believed to represent an important contributor to the global COVID-19 pandemic, especially as countries attempt to spur economic activity (2). In a significant proportion of infected individuals, the disease manifests itself with mild or no symptoms (3), and managing asymptomatic carriers, so-called “silent spreaders,” represents a significant challenge in controlling the pandemic (4). This concern is heightened by findings suggesting that SARS-CoV-2 transmission rates are similar in symptomatic and asymptomatic infected individuals (5–7). Workplace transmission from SARS-CoV-2 carriers with mild or no symptoms therefore is a potentially important mode of spreading the highly communicable disease and represents a significant occupational health risk. Infected workers subsequently are susceptible to transmitting the virus further to household members. When workplace COVID-19 acquisition occurs, standard protective measures require all employees who have been in contact with the infected individual to self-quarantine for 2 weeks. This can effectively close units and businesses even though a proportion of the isolated workers may not have contracted the virus. Based on this rationale, the development and assessment of measures to effectively control SARS-CoV-2 transmission in the workplace are highly significant and necessary to provide a safe occupational environment.

In a 15 May 2020 interview, James W. Curran, Dean, Rollins School of Public Health at Emory University, stated, “Accurate surveillance is the conscience and guidepost for public health” (8). The current clinical study builds on this foundational principle and consists of longitudinal and intensive characterization of COVID-19 prevalence and incidence at the Oak Crest Institute of Science (Oak Crest), a nonprofit science research organization in Southern California. The two primary study objectives were to (i) characterize the rate of COVID-19 acquisition in a small cohort of workers interacting on a daily basis in the workplace and (ii) determine the utility of these data in managing workplace COVID-19 exposure, minimizing further spread as per public health advisories. Exploratory aims include characterizing SARS-CoV-2 transmission between Oak Crest employees and their household members and measuring the viral dynamics in infected individuals from the study cohort.

## RESULTS

### Study participants.

A total of 54 subjects participated in the study over the first 3 months, 27 Oak Crest employees, students, and volunteers and 27 household members. Retention of study subjects was 100%. The corresponding demographics are presented in Table 1.

**TABLE 1:** Demographics of study participants

Characteristic	Value for:	
	Oak Crest employees	Household members
No. of participants enrolled	27	27
Age (yrs), median (range)	39 (21–68)	40 (11–80)
Female, no. (%)	13 (48)	16 (59)
Male, no. (%)	14 (52)	11 (41)
Race and ethnicity, no. (%)		
Black or African-American	0	0
White	23 (85)	25 (93)
Hispanic	10 (37)	14 (52)
Non-Hispanic	13 (48)	11 (41)
Asian	2 (7)	2 (7)
Other	2 (7)	0

### Sample collection efficiency.

**FIG 1:**
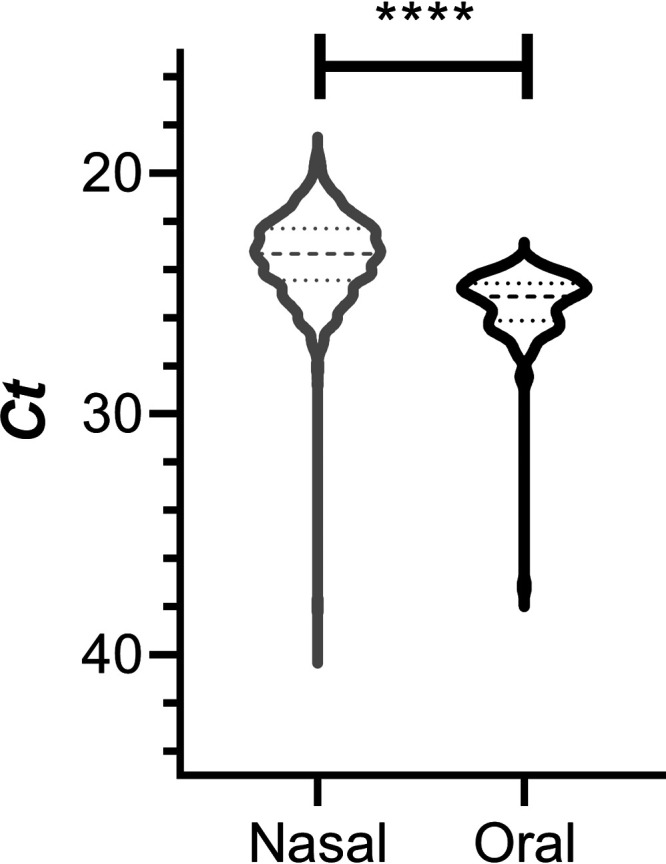
Distribution of *RP* gene transcript *C_T_* values over the 3-month clinical study. Shown is a violin plot comparing the *C_T_* values for nasal (gray) and oral (black) swab samples. Broken horizontal lines represent medians (dashed) and quartiles (dotted). Comparison of the nasal and oral swab group *C_T_* values using an unpaired, parametric, two-tailed *t* test showed that the groups were significantly different (****, *P < *0.0001).

The majority of clinical specimens consisted of nasal swab samples. Only 3 of the 54 participants (5.6%) could not tolerate the nasal swab procedure and self-collected oral samples instead. A total of 942 samples were analyzed in the 3-month study. Figure 1 compares the distribution of *RP* gene transcript cycle threshold (*C_T_*) values (i.e., indication of host RNA recovered) for the nasal (*n *= 831 [88.2%]) and oral (*n *= 111 [11.8%]) samples. The median (interquartile range [IQR]) values were as follows: nasal swab, 23.3 (22.3 to 24.4), and oral swab, 25.1 (24.6 to 26.1). The 2 populations were significantly different (*P <* 0.0001), with lower observed median *C_T_* values (i.e., more host RNA collected) for the nasal swab group. Additional results pertaining to the comparison of swab types are provided in the supplemental material.

### RT-qPCR measurement reliability.

Analysis of data from the study did not reveal any demonstrated false-negative or false-positive results from the reverse transcription-quantitative PCR (RT-qPCR) measurements. The lower limit of detection of the SARS-CoV-2 *N* gene transcript fragment RT-qPCR assay was 10 RNA copies per reaction. All positive results were confirmed by retesting on multiple, successive occasions (see below). Any false-negative results would need to have been for asymptomatic individuals who did not infect other employees or household members. A probabilistic analysis on an employee with 12 consecutive false-negative results afforded a *P* value of <0.000000001 (*P =* 1.81 × 10^−24^), assuming a 99% sensitivity and 95% specificity. If sensitivity analysis were to reduce the sensitivity and specificity to 0.90 for both, the upper probability bound over the same prevalence rates for 12 consecutive false-negative results is a *P* value of 2.3 × 10^−12^. The probability bound for a participant to have 3 consecutive false-negative results calculated over a wide range of prevalence values (0.005 to 0.5) is a *P* value of 0.0000114.

There were 5 invalid measurements in the first three study days attributed to lack of familiarity with the sampling protocol, and therefore, these were not included in the analysis. An additional 6 invalid samples were obtained during the remaining study period, accounting for 0.63% [6/(6 + 948)] of total measurements.

There were a total of 3 inconclusive measurements (0.32%) over the course of the study, with the following caveats: the inconclusive results did not include those obtained for the 2 participants who tested positive for SARS-CoV-2 RNA (see below), nor did they include 9 inconclusive results obtained on 2 study days (3 and 5 June) for participants who repeatedly tested negative for SARS-CoV-2 RNA. The high number of inconclusive measurements obtained on those 2 consecutive sampling days were attributed to reagent trace contaminants that resulted in erroneous, high *C_T_* value (i.e., low RNA copy number equivalent) background noise for amplicons obtained with the N1 or N2 primer.

### Positive participant RT-qPCR results.

**FIG 2:**
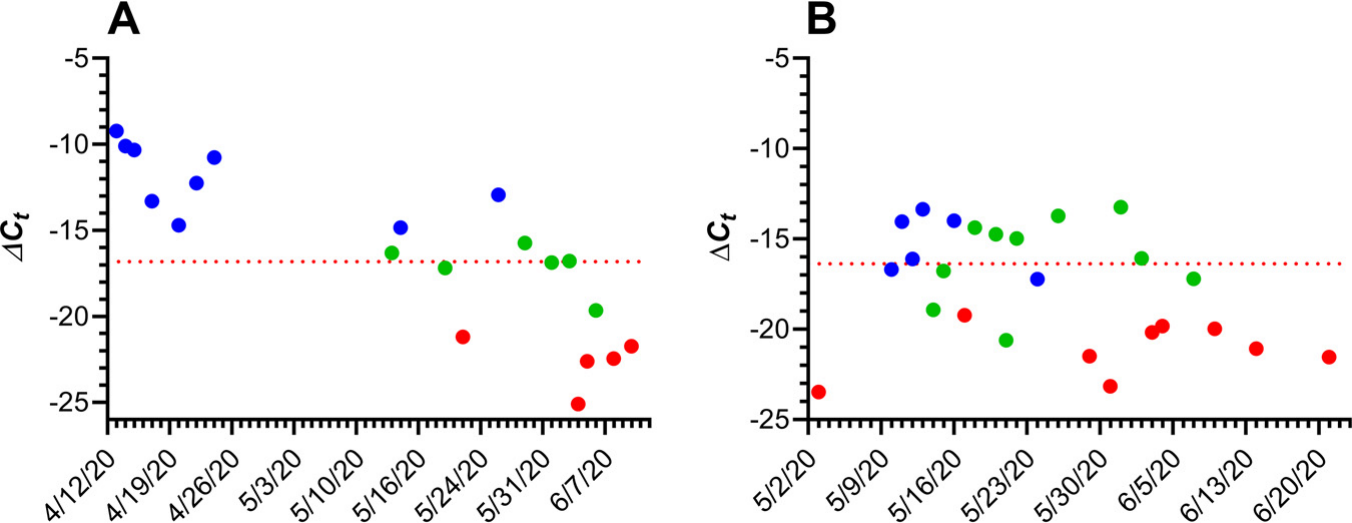
Longitudinal Δ*C_T_* values for two subjects testing positive for SARS-CoV-2 RNA (nasal swabs) during the course of the 3-month study. Blue, positive; green, inconclusive; red, negative. In cases when the measured fluorescent signal for N1 and/or N2 primers did not exceed the background level at the end of the run, the *C_T_* value was set to 46. The horizontal red broken line represents the average boundary between positive (higher Δ*C_T_* values) and negative (lower Δ*C_T_* values) based on the mean *RP* gene transcript *C_T_* for the subject over that time frame and N1 and N2 primer *C_T_* values of 40. (A) RT-qPCR results from subject 39, a recovering clinically COVID-19-diagnosed household member; the horizontal line shows a Δ*C_T_* of −16.83. (B) RT-qPCR results from subject 31, a COVID-19 asymptomatic employee, the horizontal line shows a Δ*C_T_* of −16.43.

Two participants were found to be positive for SARS-CoV-2 RNA over the course of the study (Fig. 2).

In the participant’s clinical diary, subject 39 (age, over 50 years; further demographics not disclosed for the purposes of confidentiality) described potential SARS-CoV-2 exposure on 12 March, with the onset of COVID-19 symptoms on 16 March that persisted until 31 March. During that period, the participant was self-isolated without hospitalization, and on 1 April, the participant tested positive for SARS-CoV-2 by a clinical RT-qPCR (oral swab). On 11 April, the participant enrolled in our clinical study. The last nasal swab test that met the CDC criteria for a positive result was on 26 May, indicating that the participant was COVID-19 positive for at least 71 days (16 March to 26 May). Oral (14 April) and stool (14 and 15 April) samples were negative for SARS-CoV-2 RNA.

Subject 31 (age, over 50 years; further demographics not disclosed for the purposes of confidentiality) is an employee who accessed the facility only once per week and therefore was tested weekly. The test results went from negative (up to 3 May) to positive for SARS-CoV-2 RNA (10 May) and remained positive for 14 days (until 24 May). The participant was asymptomatic during that period. Saliva (12, 13, 14, and 15 May) and stool (13, 14, 15, 16, 17, and 18 May) samples were either negative or inconclusive for SARS-CoV-2 RNA.

### Serological test results.

**FIG 3:**
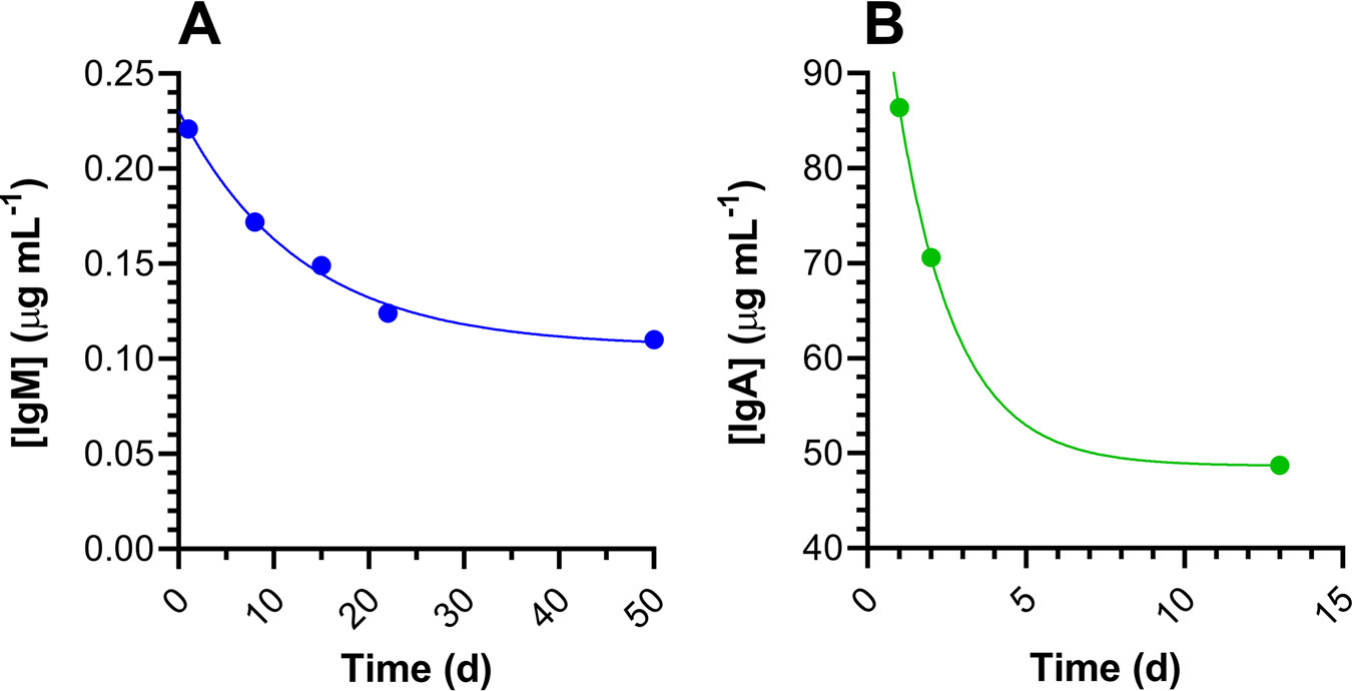
Longitudinal receptor binding domain protein (RBD) serum antibody concentrations for study participants 18 and 31 (first analysis set to day 1). (A) Serum IgM concentrations for subject 18, an employee who experienced symptoms consistent with COVID-19 that resolved just prior to the start of the study but who tested negative for SARS-CoV-2 RNA in nasal swab samples. The line shows one-phase exponential decay least-squares fit (*R*^2^ = 0.995) with a half-life of 8.8 days (d). (B) Serum IgA concentrations in subject 31, an employee who was asymptomatic but tested positive for SARS-CoV-2 RNA in nasal swab samples. The line shows one-phase exponential decay least-squares fit (*R*^2^ = 1.000) with a half-life of 1.3 days.

**FIG 4:**
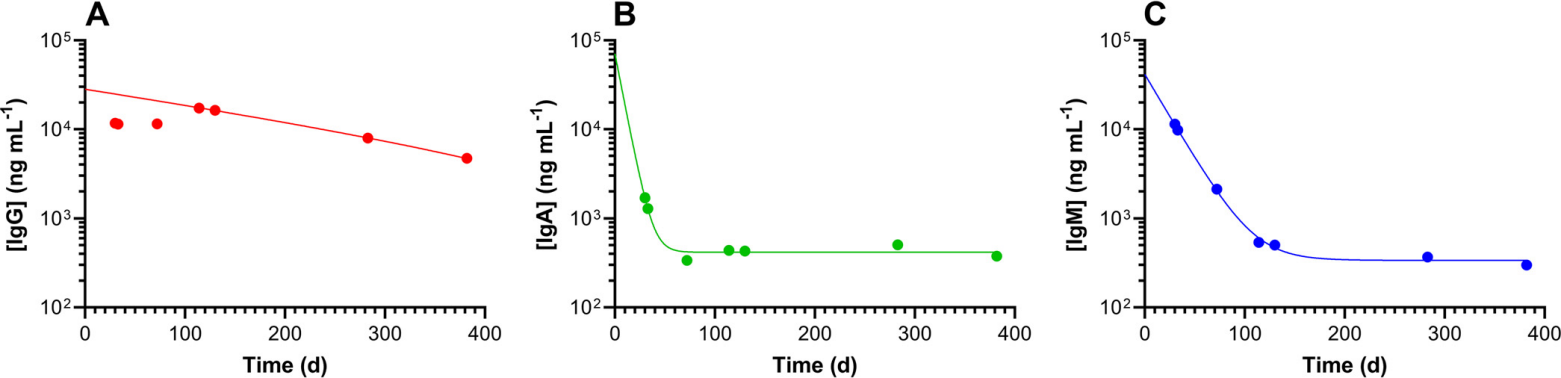
Longitudinal RBD serum antibody concentrations for study participant 39, a household member recovering from clinically diagnosed COVID-19, spanning more than 1 year (first analysis set to day 1). (A) IgG; the line shows one-phase exponential decay least-squares fit (*R*^2^ = 1.000) with a half-life of 183.4 days (note that the fit starts at the maximum concentration [*C*_max_], 114 days). (B) IgA; the line shows one-phase exponential decay least-squares fit (*R*^2^ = 0.990) with a half-life of 5.2 days. (C) IgM; the line shows one-phase exponential decay least-squares fit (*R*^2^ = 1.000) with a half-life of 15.7 days.

A total of 33 serum samples collected between 27 March and 26 May 2020 were analyzed for virus-specific IgM, IgG, and IgA concentrations as described above. The results are summarized in Table 2 and Fig. 3. Virus-specific IgM, IgG, and IgA concentrations in serum samples from subject 39 were measured for over 1 year (Fig. 4). Other than subject 39, only one participant had detectable serum IgG concentrations (collected on 15 May; 0.12 μg ml^−1^).

**TABLE 2:** Summary of RBD antibody concentrations in participant serum samples at baseline, 27 March to 3 April 2020 (*n *= 16), and for subject 39 over the 3-month study period

Parameter	Concn (μg ml^−1^)		
	IgM	IgG	IgA
LLD^*a*^	0.0049	0.0017	0.0017
% > LLD^*b*^	56	0	6
Median (IQR), baseline^*c*^	0.39 (0.24–0.96)	NA^*e*^	0.49
Median (range) for subject 39^*d*^	9.83 (2.12–11.4)	11.5 (11.5–11.7)	1.29 (0.34–1.70)

a Assay limit of detection (LLD) determined at 1:100, 1:40, and 1:20 dilutions. The LLD is presented as the diluted concentration.

b Data represent proportions of samples that contained detectable antibody concentrations.

c Interquartile range (25th to 75th percentile).

d Because of the high antibody concentrations measured in multiple samples, the data for subject 39 are presented separately.

e NA, not applicable.

### Model results of local study impacts.

The predictive model for cases, hospitalizations, and deaths described earlier was run for California with prevalence levels seen on 23 March 2020 and on 7 July 2020 in Los Angeles County. Given 27 employees and 27 household contacts with the demographic characteristics of the participants (Table 1) from the current study and without surveillance interventions, the model produced an expectation that up to 7 employees (95% confidence interval [CI] = 3 to 10) would have become infected, with at most 1 of them requiring hospitalization and 0 deaths. The lack of deaths stems from the population demographic tending toward younger persons and improved in-hospital treatment procedures at the time of writing.

### Practical study considerations.

A number of policies and procedures were instituted during the study, as described below. Some were part of the original protocol, and others were added based on practical considerations. While working on site at Oak Crest, all individuals practiced social distancing and hand hygiene and wore face coverings when in close proximity to other employees on Mondays (see below); testing negated the need for mandatory face masks on other days. Outside of work, employees and their household members were encouraged to strictly adhere to current public health advisories regarding gathering in groups, wearing face coverings, and social distancing.

Participants who tested positive for SARS-CoV-2 RNA received referrals for testing and recommendations (e.g., self-quarantine) based on the current, preferred public health requirements. Symptomatic participants, even if testing negative for SARS-CoV-2 RNA by RT-qPCR, would have been asked to return home (none were reported in the study), self-quarantine at a minimum until symptoms resolved, and contact their health care provider about confirmatory clinical testing and symptom management. Participants testing positive for SARS-CoV-2, even if asymptomatic, were informed as soon as possible and asked to go home, self-quarantine, and contact their health care provider or the Department of Public Health (DPH) about confirmatory testing, treatment, and quarantine recommendations. Participants also were given educational information to guide other household members of potential risk and some of their options. Any employee who was asked to self-quarantine, or did not choose to participate in the study, worked remotely as much as possible and continued to receive full pay.

Participants who tested negative and obtained a subsequent invalid test result were asked to wear protective face covering (surgical masks were provided by Oak Crest if needed) until they could be retested. Initially, participants who tested negative and obtained a subsequent inconclusive test result were sent home immediately and told to self-quarantine until they could be retested (i.e., new swab sample collected and analyzed). As the study progressed, that protocol was modified to be the same as for an invalid result (i.e., the employee was asked to wear protective face covering rather than being sent home for self-quarantine), assuming that previous test results were negative. The longest period between tests was Friday to the following Monday (3 days). Starting in May, all employees were required to wear protective masks on Monday mornings until the test results were finalized and communicated. The same procedure was instituted for 3-day weekends when there was an additional day between tests. Employees who missed 1 day of testing were required to wear a protective mask until they were retested. Employees who missed more than 1 day of testing were permitted to reenter the facility only after a new sample tested negative.

## DISCUSSION

The current, ongoing clinical study has two primary aims: (i) to characterize the rate of SARS-CoV-2 acquisition in a small cohort of participants interacting on a daily basis in the workplace and (ii) to determine the ability of these data to manage workplace SARS-CoV-2 exposure and consequences, minimizing further spread as per public health advisories. Workplace study participants include staff, students, and volunteer researchers, referred to as “employees.” Family members and housemates also were recruited leading to a subcohort referred to as “household members.” Related exploratory study goals include characterizing the rate of SARS-CoV-2 acquisition in employee and household members, quantifying antibody-specific responses in blood at baseline (previously exposed) and while on study (to capture asymptomatic/presymptomatic, newly infected individuals), and characterizing viral shedding parameters in saliva and stool samples.

Clinical SARS-CoV-2 RNA test kits are required to have received Emergency Use Authorization (EUA) by the FDA and are processed in a laboratory certified according to the Clinical Laboratory Improvement Amendments (CLIA). Such kits have been a rare resource during this clinical study and, consequently, were not available for investigative purposes. Our clinical study used research reagents to achieve outcomes but did not interfere with the availability of reagents in short supply for clinical diagnosis. The RT and amplification kit (TaqPath 1-step RT-qPCR master mix) and primers have not been in short supply. The employed RNA extraction kit (Quick-RNA viral kit) did not have FDA EUA in March 2020 and has been readily available since then. Nasopharyngeal swabs were in short supply early in the study, requiring the use of existing, in-house stocks until they became more readily available starting in May 2020 (see Fig. S2 in the supplemental material). The various swab types performed similarly and met the study criteria. We analyzed approximately 1,000 samples over the course of the 3-month study, compared to over 37.2 million clinical tests reported for the United States over a similar period according to the Centers for Disease Control and Prevention (9).

10.1128/mSphere.00542-21.4 FIG S2 Distribution of *RP* gene transcript *C_T_* values over the 3-month clinical study. Shown are box plots of *C_T_* values from nasal and oral swab samples for each study day. The box extends from the 25th to 75th percentiles, with the horizontal line in the box representing the median; whiskers represent the 10th to 90th percentiles. Colors are representative of different nasopharyngeal swab types: blue, model 220246, BD Diagnostics; red, model 25-806 2PD, Puritan Medical Products; green, model 25-3406-H, lot 2937, Puritan Medical Products; orange, model 25-3000-H, Puritan Medical Products; and magenta, model 25-3406-H, lot 7221, Puritan Medical Products. Download FIG S2, TIF file, 0.5 MB. Copyright © 2021 Gunawardana et al. 2021 Gunawardana et al. https://creativecommons.org/licenses/by/4.0/ This content is distributed under the terms of the Creative Commons Attribution 4.0 International license.

The study was designed to be efficient in terms of manpower commitment and protecting the study team from exposure to SARS-CoV-2 during sampling. Employees were trained in self-sample collection while isolated in their vehicles 3 times per week (Monday, Wednesday, and Friday) between 8:30 and 9:15 a.m. with support from the study team. The samples (typically 23 to 28 per day) were processed the same day, with results available by early afternoon. A team of 4 researchers (2 handling samples and 2 observing for quality assurance [Fig. S1B]) performed the RNA extraction and loading of the 96-well plate for RT and cDNA gene fragment amplification, typically completed within 90 min. Additional labor included quality assurance and subsequent data analysis. These resource commitments made the study feasible to continue indefinitely until recovery from the COVID-19 pandemic allows for a safe workplace.

10.1128/mSphere.00542-21.3 FIG S1 Photographs documenting sample collected and analysis in the OCIS-05 clinical study. (A) Sample collection from participant who previously self-collected a nasal swab specimen in their vehicle. (B) Extraction of SARS-CoV-2 RNA by study team. Download FIG S1, TIF file, 1.6 MB. Copyright © 2021 Gunawardana et al. 2021 Gunawardana et al. https://creativecommons.org/licenses/by/4.0/ This content is distributed under the terms of the Creative Commons Attribution 4.0 International license.

During the course of the study, one employee (subject 31) became SARS-CoV-2 RNA positive for ca. 2 weeks (Fig. 2B). The viral RNA copy number (median, 13.1 copies per swab; IQR, 10.9 to 18.8 copies per swab) remained low throughout, and the subject did not report any COVID-19 symptoms. Blood draws on 14, 15, and 26 May from the subject produced serum samples that were below the analytical assay limit of detection (LLD) for 2 of the measured anti-SARS-CoV-2 monoclonal antibodies (IgM and IgG). However, anti-receptor binding domain protein (anti-RBD) IgA concentrations were detectable in all samples and decayed rapidly (half-life [*t*_1/2_] = 1.3 days [Fig. 3B]).

It is becoming increasingly evident based on literature reports that a significant proportion of individuals with positive SARS-CoV-2 RT-qPCR results remain asymptomatic or minimally symptomatic (5, 10–13) yet still contagious (5, 13). However, ours is the first report on longitudinal analysis of an asymptomatic individual who maintained low SARS-CoV-2 RNA copy numbers and did not appear to trigger a traditional host immune response. This result is not entirely unexpected, as antibody responses are not detectable in all COVID-19 patients, especially those who experience low-grade symptoms (10, 14).

One household member (subject 39) was symptomatic and clinically diagnosed with COVID-19 by RT-qPCR and began participating in the study during convalescence after symptoms resolved (Fig. 2A). The subject developed a robust immune response in terms of anti-SARS-CoV-2 IgM, IgG, and IgA serum concentrations (Table 2 and Fig. 4) that persisted for over 1 year from the onset of symptoms, allowing decay half-lives for all three antibody types to be calculated. The intense longitudinal RNA sampling provided a rare insight into the viral dynamics of an individual recovering from COVID-19 over a 3-month time frame. Subject 39 remained positive for viral RNA for at least 71 days, one of the longest periods of SARS-CoV-2 shedding reported at that time. Little is known about this potential subpopulation of individuals with persistent, long-term viral recalcitrance.

Intermittent news reports suggest that some convalescing COVID-19 patients who have tested negative for SARS-CoV-2 RNA by RT-qPCR may retest positive later in recovery, prompting speculation that some individuals are vulnerable to reinfection. Others hypothesize that positive retests are the result of noninfectious, residual viral fragments. Unfortunately, many of these reports are anecdotal and not scientifically controlled. Scientific studies on the alleged SARS-CoV-2 reinfection phenomenon are scarce. There are isolated case reports describing a situation in which a single COVID-19 patient was discharged, in part because of two consecutive negative SARS-CoV-2 RT-qPCR clinical results, but retested positive for SARS-CoV-2 RNA during convalescence (15–19), including a 71-year-old woman who tested positive for 60 days from the onset of symptoms (55 days from her first positive test) (20). Other accounts detail similar results for small cohorts of 2 or more individuals (21, 22). In a clinical study involving 66 patients who recovered from COVID-19, 11 (16.7%) retested positive for viral RNA in stool samples during convalescence (23). A cohort of 86 COVID-19 patients was retested by RT-qPCR less than 28 days after self-reported symptom resolution. Eleven subjects (13%) were still diagnosed as positive by RT-qPCR at a median of 19 days (range, 12 to 24 days). An et al. followed 262 COVID-19 patients for 2 weeks following discharge and found that 14.5% tested positive for SARS-CoV-2 RNA, suggesting that this subset may be carriers of the virus (24). In a similar study, Huang et al. found that out of 414 recovering COVID-19 patients, discharged and subsequently quarantined, 69 (16.7%) retested positive for SARS-CoV-2, 13 with 2 readmissions and 3 with 3 readmissions (25), suggesting that in some cases the virus was replication competent. The above-mentioned cases are distinct from so-called “long-haul COVID,” in which patients who recovered from the acute phase of COVID-19 continue to experience symptoms for long periods (>100 days) (26).

All Oak Crest employees with the exception of subject 31 remained negative for SARS-CoV-2 RNA over the course of the 3-month study. In our hands, the RT-qPCR appeared to provide reliable results, with no known false positives or negatives. With the exception of subjects 31 and 39, inconclusive measurements (i.e., only one of the two oligonucleotide probes targeting the viral nucleocapsid protein gene transcript fragment met the assay threshold for positivity) made up a small fraction (0.32%) of the test results. We contend that in the context of a person developing or recovering from COVID-19 infection, inconclusive SARS-CoV-2 RNA measurements can define the transition phase between positive and negative, as supported by Fig. 2. Keeping in mind data published regarding the potential reinfectivity of SARS-CoV-2, subjects 31 and 39 were continuously retested, even following occasions where subjects received 2 consecutive negative results. Scattered inconclusive results accompanying days of high *C_T_* values for subjects 31 and 39 provide insight into the potential for reinfection and speak to the sensitivity of our assay. This study is especially useful in distinguishing between cases that appear to be true reinfections and those that could be classified by the transition from a positive to a negative result.

Unexpectedly, 9 of 16 employees (56%) tested positive for anti-SARS-CoV-2 antibodies, mostly IgM, at baseline between 27 March and 3 April (Table 2), suggesting possible exposure to the virus prior to the start of the clinical study. Many of these participants reported suffering from symptoms consistent with COVID-19—thought to be a virulent flu strain at the time—in the first half of February. However, none of these serum samples had detectable concentrations of IgG, which has the longest half-life of the three measured virus-specific antibodies (Fig. 4) (27). The findings remain unexplained and could be due to cross-reactivity in the IgM assay. Subject 18, a self-quarantined employee who had just recovered from suspected COVID-19 (based on symptomology) at the start of the study, repeatedly tested negative for SARS-CoV-2 RNA but tested positive for IgM antibodies that rapidly declined (*t*_1/2_ = 8.8 days [Fig. 3A]). No IgG or IgA antibodies were detected in serum samples from this participant.

There are some caveats to interpreting our antibody data. We measured only IgM, IgG, and IgA against RBD and not the whole spike or E proteins, as discussed in more detail elsewhere (27). The IgM assay is subject to greater uncertainty than the IgA and IgG assays at low concentrations (ca. 0.25 μg ml^−1^ or lower), and results need to be interpreted with caution. However, all serum samples were analyzed at three levels of dilution (1:100, 1:40, and 1:20), and only detectable concentrations in at least 2 of the 3 diluted samples are reported.

The clinical study described here has met its goals to date by providing a safe work environment for employees of a small academic institute, at a time when the United States was experiencing alarming expansion of the pandemic. The testing model constitutes a validated, practical framework that could be adopted by other small organizations for this and future respiratory virus pandemics. The ability to perform routine, frequent COVID-19 testing not only has allowed us to responsibly maintain a productive work environment during the pandemic but also has provided continuous reassuring data for the participating households, many including vulnerable individuals. This had the important mental health benefits of reducing anxiety and providing a sense of normalcy in the workplace (i.e., safe zone) during an acutely stressful period.

The clinical study’s success in preventing workplace SARS-CoV-2 transmission can be assessed through a predictive analysis. Modeling local study impacts predicted that without our intervention, up to 7 employees or household contacts could have become infected with SARS-CoV-2 by 7 July 2020. Our finding that only two subjects contracted COVID-19, one prior to the start of the study, suggests that workplace disease surveillance based on frequent, longitudinal testing combined with recommended public health practices (social distancing, frequent hand sanitizing, and mask wearing on selected days), when feasible, can be effective at creating a safe zone or bubble preventing COVID-19 from entering into the workplace.

Our simulations predicted an important, local public health benefit from our scalable surveillance plan. Similar approaches were adopted subsequently in the summer of 2020 by professional sports leagues (e.g., the National Hockey League), the entertainment industry, and others to support responsible resumption of their activities. The study also generated important, new scientific data on the SARS-CoV-2 host dynamics enabled by its longitudinal, intense sampling design. Our clinical study represents a powerful example on how an innovative public health initiative can be dovetailed with scientific discovery.

## MATERIALS AND METHODS

### Ethics statement.

All human research under OCIS-05, “Longitudinal Characterization of COVID-19 Prevalence and Incidence in a Small Working Institution with Both Public Health and Diagnostic Aims,” was approved by the Aspire institutional review board (IRB) (Aspire study number 1281548) and conducted according to the Declaration of Helsinki. All 54 study participants provided written informed consent or assent.

### Clinical study design.

The clinical study was initiated on 23 March 2020 and is ongoing at the time of writing. Results from the first 3 months of testing (23 March 23 to 22 June 2020) are presented here. All employees, students, and volunteers at Oak Crest (https://www.oak-crest.org/), a small nonprofit academic science research organization located in Monrovia, CA, were asked to participate in the prospective, longitudinal, observational study designed to last 12 weeks, or longer. Those choosing not to participate had no negative employment or finance-related consequences but were asked to work from home exclusively. Household members from the above-described study population also were invited to participate in the study. At study onset, up to 30 Oak Crest employees and up to 60 household members were anticipated to participate. Additional details are provided in the supplemental material.

Test results for nasal or oral swabs collected in the morning typically were available early in the afternoon of the same day. When a participant tested positive or the results were inconclusive, testing was repeated for confirmation or performed at increased frequency (daily when feasible) until the participant had repeated negative results. Participants were advised in the informed consent and, again, when informed of research test results that these results were research based and not intended for clinical diagnosis. Participants received referrals for testing and recommendations (e.g., self-quarantine) based on the current, preferred public health requirements at the time.

### Sample collection.

Nasal sample collection was overseen by two researchers equipped with personal protective equipment (PPE) in the Oak Crest parking lot, starting at 8:30 a.m. Typically, 30 samples or fewer were collected in under 45 min. Additional details are provided in the supplemental material.

Under certain circumstances, study participants self-collected swab samples at home and placed the swab tip in a microcentrifuge tube (1.5 ml) containing DNA/RNA Shield (300 μl; Zymo Research, Tustin, CA). The samples were stored and transported at room temperature or 4°C and processed within 60 h. Control experiments showed that RNA integrity was maintained under these conditions.

Optional saliva samples were self-collected in Falcon tubes (50 ml) at the participant’s home or in their sealed vehicle, and stool swabs were collected at the participant’s home. Specific written instructions were provided to participants opting to provide these specimens. Blood (5 to 8 ml, ×2) was collected by a licensed phlebotomist using Vacutainer (Becton, Dickinson and Company, Franklin Lakes, NJ) tubes for serum (spray-coated silica) and plasma (spray-coated K_2_EDTA) in the Oak Crest parking lot, while the participant remained comfortably seated in their vehicle.

### Chemicals and reagents.

All reagents were obtained from Sigma-Aldrich (St. Louis, MO), unless otherwise noted.

### RNA extraction.

RNA was extracted from swab specimens in the collection tubes using the Quick-RNA viral kit (R1036; Zymo Research) according to the manufacturer’s instructions, consistent with the CDC COVID-19 testing kit guidelines (28). The kit is designed for the rapid isolation of high-quality RNA from a range of biological sources, including cellular suspensions.

### RT-qPCR analysis.

A one-step reverse transcription (RT) and quantitative PCR (qPCR) was performed using the TaqPath 1-step RT-qPCR master mix (Thermo Fisher Scientific, Waltham, MA) in a 20-μl final reaction volume per the manufacturer’s instructions. Three target sequences were amplified simultaneously (29) in accordance with CDC diagnostic COVID-19 testing guidelines (28): two SARS-CoV-2 nucleocapsid protein (*N*) gene transcript fragments (N1 and N2) and one human RNase P (*RP*) gene transcript fragment (RP). The primer/probe sequences are as described elsewhere (28) and were obtained from Integrated DNA Technologies (10006606 and 10006713; Coralville, IA). RNA standards for SARS-CoV-2 nucleocapsid (*N*) was obtained from Microbiologics (HE0060-100-T; St. Cloud, MN) and served as positive controls. Confluent Caco-2 cells were used as a human specimen control (HSC) according to CDC guidelines (28).

Every panel consisted of one 96-well plate that accommodated 28 clinical specimens, each with three PCR probes (28 × 3 = 84 wells), HSC (1 × 3 = 3 wells), SARS-CoV-2 RNA standard as a positive control (1 × 3 = 3 wells), and a no-template control (1 × 3 = 3 wells), leaving 3 wells that were used for additional validation (e.g., naive swab). This panel configuration meets or exceeds the CDC guidelines (28).

Ninety-six-well PCR plates were prepared using reagents on ice and centrifuged at 1,000 rpm for 1 min at room temperature. The plates were run on a CFX96 Touch real-time PCR detection system (Bio-Rad Laboratories, Inc., Hercules, CA) using the following cycling conditions: stage 1, 25°C for 2 min (1 cycle); stage 2, 50°C for 15 min (1 cycle); stage 3, 95°C for 2 min (1 cycle); stage 4, step 1, 95°C for 3 s, and step 2, 55°C for 30 s, with a loop of 45 cycles of steps 1 and 2 (total, 46 cycles per assay). Stages 1 and 2 were reverse transcription, stage 3 was inactivation, and stage 4 was amplification.

The normalized SARS-CoV-2 RNA copy number, Δ*C_T_* was calculated according to equation 1:

(1) $$\Delta C_{T}=C_{T}^{\mathrm{RP}}\;-\;C_{T}^{N}$$

where *C_T_^RP^* is the *C_T_* value for the *RP* gene transcript in the sample and *C_T_^N^* is the mean *C_T_* for corresponding two SARS-CoV-2 *N* gene transcripts (N1 and N2).

### SARS-CoV-2 *N* gene RT-qPCR probe calibration.

Calibration plots of *C_T_* versus RNA copy number were generated for every batch of TaqPath 1-step RT-qPCR master mix kits. Predetermined amounts of RNA standard for SARS-CoV-2 *N* gene transcript fragments (see above) were serially diluted in nuclease-free water (Promega Corporation, Madison, WI). In a typical experiment, eight calibration standards were used, spanning the RNA copy number range from 0.15 to 1.5 × 10^6^. The samples (20 μl) were analyzed (N1 and N2 probes) as described above.

### Assay result interpretation.

The diagnostic panel result interpretation followed CDC guidelines (28). Specimens with a *C_T_* less than 40 for N1, N2, and RP were considered positive. Samples with a *C_T_* greater than 40 for N1 and N2 but less than 40 for RP were considered negative. Samples with a *C_T_* for either N1 or N2 less than 40 (i.e., only one of the two probes resulted in a *C_T_* less than 40) and a *C_T_* for RP less than 40 were considered inconclusive. Samples with a *C_T_* for RP greater than 40 were considered invalid.

### Swab assessment.

A range of collection swabs were used in the clinical study due to supply shortages (see Results). All swabs were evaluated for fungal and bacterial contamination by qPCR using published methods (30, 31), with additional details provided in the supplemental material.

### Quantification of serum IgG, IgM, and IgA against SARS-CoV-2.

### (i) Production of soluble SARS-CoV-2 spike receptor binding domain protein.

SARS-CoV-2 spike receptor binding domain protein (RBD) was produced by transient transfection of HEK-293F cells with plasmid pCAGGS (32, 33), containing the SARS-CoV-2 Wuhan-Hu-1 spike glycoprotein gene RBD with C-terminal hexahistidine tag (generously provided by F. Krammer). Transfection was carried utilizing the Expi293 expression system (Gibco, Thermo Fisher Scientific) following the manufacturer’s instructions. RBD was purified by affinity chromatography utilizing nickel-nitrilotriacetic acid (Ni-NTA) agarose (Thermo Fisher Scientific), and RBD purity was assessed by SDS-PAGE.

### (ii) ELISA for antibodies against RBD.

Antibody responses against RBD were measured in a modified enzyme-linked immunosorbent assay (ELISA) based on a protocol by Krammer’s group (32, 33) (see the supplemental material for more details).

### Model analysis.

A COVID-19 infection model that combines traditional susceptible, exposed, infected, and recovered (SEIR) compartmental models with Bayesian time series and modern machine learning was used for model analysis (34). This model fuses three methods to provide accurate predictions of case counts and hospitalizations. Briefly, a Bayesian time series model was fitted to the velocity (first derivative) of the cumulative case counts for each location, such as California, or a specific county, zip code, or even workplace and incorporated prior information such as local interventions to obtain the posterior distribution of the trajectory of the cases. This was then fed into the compartmental model to predict deaths using the random forest algorithm trained on COVID-19 data and population-level characteristics such as age, expected proportion of comorbidities, sex, and race/ethnicity. This yielded daily projections and interval estimates for cases and deaths for each location. The approach combined the strengths of traditional epidemiologic compartmental models with curve-fitting statistical models and modern machine learning.

### Data analysis.

Data sets were analyzed using GraphPad Prism (version 8.4.3; GraphPad Software, Inc., La Jolla, CA). Statistical significance was defined as two-sided *P *value of <0.05. The unpaired, parametric, two-tailed *t* test was used to compare nasal and oral swab *RP* gene transcript *C_T_* values. Positivity only was defined from qPCR results, not by analysis of blood/serum samples, and hence, IgG, IgA, and IgM concentrations are not included in probability estimates.

The probability of multiple, sequential false-negative SARS-CoV-2 results was estimated using traditional probability theory. Each serial test was assumed to be independent, as the probability of a test result outcome was not dependent on the result of the previous test. The sensitivity of the qPCR test was assumed to be 0.99 and the specificity 0.95 for nasal and oral swab samples. The probability of being COVD-19 positive given a negative test result was estimated according to Bayes’ theorem, equation 2:

(2) $$P\left(D\text{|}N\right)=\frac{P(N|D)\times P(D)}{P(N)}$$

where *P*(*D|N*) is a conditional probability, the likelihood of event *D* occurring given that *N* is true; *P*(*N|D*) is a conditional probability, the likelihood of event *N* occurring given that *D* is true; and *P*(*D*) and *P*(*N*) are the probabilities of observing *D* and *N*, respectively. This probability was estimated for prevalence ranges from 0.05 to 0.5, and the greatest upper bound was taken as the probability bound to error on the side of conservativeness.

10.1128/mSphere.00542-21.1 TEXT S1 Additional experimental details pertaining to the clinical study. Download Text S1, DOCX file, 0.02 MB. Copyright © 2021 Gunawardana et al. 2021 Gunawardana et al. https://creativecommons.org/licenses/by/4.0/ This content is distributed under the terms of the Creative Commons Attribution 4.0 International license.

10.1128/mSphere.00542-21.2 TEXT S2 Additional results pertaining to the sample collection efficiency. Download Text S2, DOCX file, 0.02 MB. Copyright © 2021 Gunawardana et al. 2021 Gunawardana et al. https://creativecommons.org/licenses/by/4.0/ This content is distributed under the terms of the Creative Commons Attribution 4.0 International license.

10.1128/mSphere.00542-21.5 TABLE S1 Study evaluations; summary of the questionnaire and sample collection framework in the clinical study Table S1, PDF file, 0.1 MB. Copyright © 2021 Gunawardana et al. 2021 Gunawardana et al. https://creativecommons.org/licenses/by/4.0/ This content is distributed under the terms of the Creative Commons Attribution 4.0 International license.
