# Supplementary figures and images for: Longitudinal COVID-19 Surveillance and Characterization in the Workplace with Public Health and Diagnostic Endpoints
Source: mSphere. 2021 Jul 7;6(4):e00542-21. doi: 10.1128/mSphere.00542-21 (PMC8386432; doi:10.1128/mSphere.00542-21)

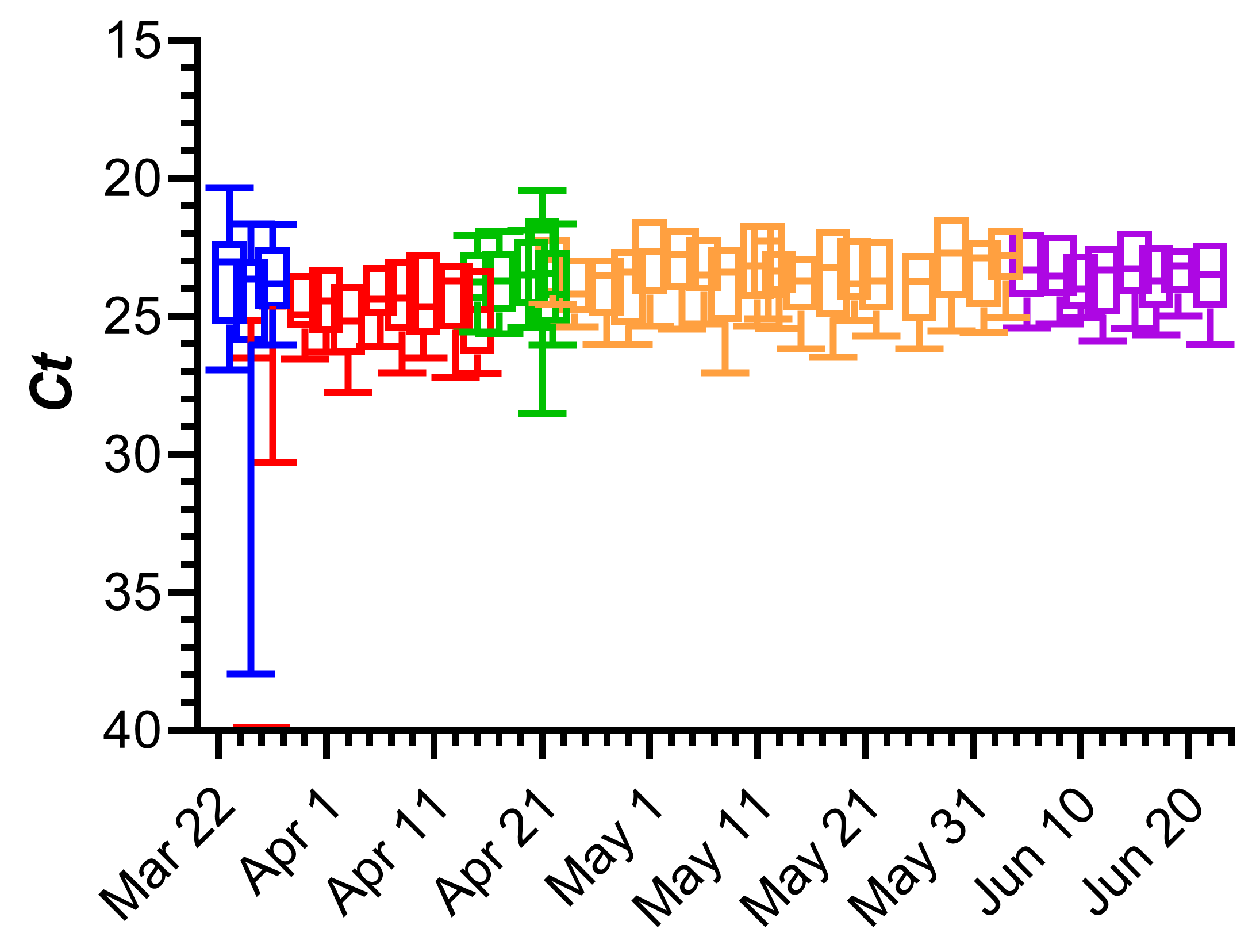

Supplement: FIG S2 [file msphere.00542-21-sf002.tif]

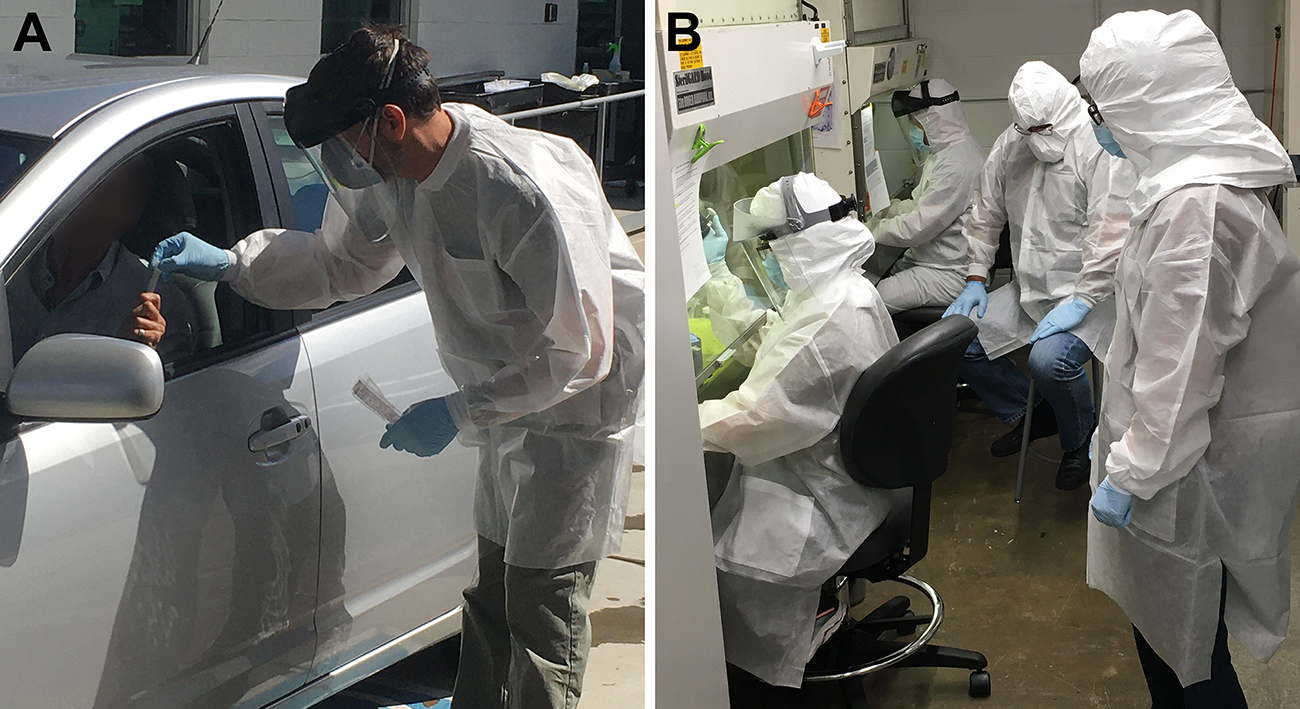

Supplement: FIG S1 [file msphere.00542-21-sf001.tif]
