# Supplementary material for: Longitudinal COVID-19 Surveillance and Characterization in the Workplace with Public Health and Diagnostic Endpoints
Source: mSphere. 2021 Jul 7;6(4):e00542-21. doi: 10.1128/mSphere.00542-21 (PMC8386432; doi:10.1128/mSphere.00542-21)
Supplement: TEXT S1 [file msphere.00542-21-s0001.docx]

**Longitudinal COVID-19 Surveillance and Characterization in the Workplace with Public Health and Diagnostic Endpoints**

Manjula Gunawardana,^1^ Jessica Breslin,^1†^ John M. Cortez, Jr.,^1†^ Sofia Rivera,^1†^ Simon Webster,^1†^ F. Javier Ibarrondo,^2^ Otto O. Yang,^2,3^ Richard B. Pyles,^4,5^ Christina M. Ramirez,^6^ Amy P. Adler,^7^ Peter A. Anton,^1^ and Marc M. Baum^1*^

^1^Department of Chemistry, Oak Crest Institute of Science, 128-132 W. Chestnut Ave., Monrovia, California, United States of America

^2^University of California, Los Angeles (UCLA), Division of Infectious Diseases, Department of Medicine, David Geffen School of Medicine at UCLA, Los Angeles, California, United States of America

^3^University of California, Los Angeles (UCLA), Department of Microbiology, Immunology, and Molecular Genetics, David Geffen School of Medicine at UCLA, Los Angeles, California, United States of America

^4^Department of Pediatrics, University of Texas Medical Branch, Galveston, Texas, United States of America

^5^Department of Microbiology and Immunology, University of Texas Medical Branch, Galveston, Texas, United States of America

^6^University of California, Los Angeles (UCLA), Department of Biostatistics, Fielding School of Public Health, UCLA, Los Angeles, California, United States of America

^7^Jumpstart Research Consulting, LLC, Santa Fe, New Mexico, United States of America

**Supplemental Material**

**Methods and Materials**

**Clinical Study Design**

Volunteers who met the inclusion criteria were asked to provide written consent (or assent in the case of minors) to participate in the study, under an institutional review board approved protocol. At baseline, participants also completed a survey that included basic demographic information (name, address, phone number, date of birth, gender, race, and ethnicity), relevant co-morbid conditions, contact with possible COVID-19 positive individuals, and recent travel history (**Supplemental Table S1**). A symptom diary also was completed (**Supplemental Table S1**). Where relevant, entries were updated weekly. The above items were the extent of personal history and demographics collected. No medical records were obtained/reviewed. Because of the nature of the study population, participants were informed in the consent form that, “Although all efforts will be made to protect your privacy and confidentiality (we will explain more below), other people in your house and at OCIS will know you are in the study. The results of your tests could become known if you have to be quarantined at home.”

The clinical study was unblinded and there were no study-specific interventions. Descriptive and summary statistics are provided for this small observational study.

**Sample Collection**

Study participants were required to wear masks, waited in their parked vehicles, and received a sterile nasopharyngeal swab (synthetic applicator tip, synthetic handle) from one of the two study researchers (**Supplemental Figure S1A**).

Due to limited availability, five different swab types/lots were used over the course of the study, in chronological order: (1) eSwab (Model 220246, BD Diagnostics, Sparks, MD); (2) sterile polyester tipped applicator (Model 25-806 2PD, Puritan Medical Products, Guilford, ME); (3) flocked collection device (Model 25-3406-H, Lot 2937, Puritan Medical Products); (4) flocked collection device (Model 25-3000-H, Puritan Medical Products); and (5) flocked collection device (Model 25-3406-H, Lot 7221, Puritan Medical Products). Prior to sample collection, the subject closed the vehicle window and self-collected one nasal specimen (one swab, both nares) according to the study instructions. An oral swab sample (gums, cheeks, and back of throat) was collected according to the study instructions *in lieu* of the nasal swab if the latter could not be tolerated. The used swab tip was placed in a pre-labeled, empty (i.e., dry) microfuge tube (1.5 mL), the swab handle broken off, and the closed tube handed to the researcher *via* the open window. If the participant was uncomfortable breaking the swab handle, the used swab was handed to the researcher who then broke it and sealed the tip in the sample tube. The sample tube was sprayed with 70% v/v isopropanol and placed on wet ice until all samples were collected.

**Swab Assessment**

The two most common swab types employed were model 25-2000-H and 25-806-2PD (Puritan, Guilford, MA). Swab batches that were found to be free of microbial contamination were evaluated for nasal sample collection efficiency. Typically, 3-6 volunteers from the clinical study self-swabbed nasally according to the study protocol with the test swabs. The swabs were analyzed as described above using the RP probe, and *Ct* values below 25 typically were indicative of efficient sample collection. Swabs from that batch then were used in the study.

**Quantification of Serum IgG, IgM, and IgA against SARS-CoV-2**

Enzyme-linked Immunosorbent Assay (ELISA) for Antibodies against RBD. In brief, 96-well microtiter plates were coated with soluble RBD protein (2 µg mL^-1^) in calcium- and magnesium-free phosphate buffered saline (PBS, Gibco). The plates were washed three times in PBS containing 0.1% Tween-20 (TPBS) and incubated with PBS containing 3% dried milk (bioWORLD, Dublin, OH) for a minimum of one hour at room temperature before removal. Participant serum was added in duplicates in three-fold serial dilutions from 1:40 to 1:1080 in PBS and incubated at room temperature for two hours. The plates were washed three times with TPBS, and the secondary antibody anti-human IgG-horseradish peroxidase (Bethyl Laboratories, Montgomery, TX) –or anti-human IgM-horseradish peroxidase, or anti-IgA-horseradish peroxidase– was added in PBS at a 1:50,000 dilution for incubation at room temperature for one hour. The plates were washed three times with TPBS, followed by addition of 100 µL TMB substrate solution (ThermoFisher Scientific) for 10 minutes at room temperature and then 100 µL sulfuric acid stop solution (ThermoFisher Scientific). The plates were read at 450 and 650 nm wavelengths on a Spark 10M microplate reader (Tecan, Baldwin Park, CA). Each plate also contained wells with the control anti-RBD monoclonal IgG antibody CR3022 (Creative Biolabs, New York, NY) plated in serial dilutions to establish a standard curve, or an IgM or IgA monoclonal antibody with the same variable region as CR3022 produced in-house.

Briefly, IgA and IgM RBD-specific control antibodies were produced by transfection of 293F cells with plasmids coding the CR3022 light chain, the J chain and the human IgA or IgM heavy chains containing the heavy variable region of CR30222 (plasmids were kindly provided by G. Alter). Five days after transfection, antibodies were purified by affinity chromatography. Purity and antibodies chain stoichiometries were assessed by SDS-PGE under reducing and non-reducing conditions.

Optical density values from participant serum were compared to the standard curve to extrapolate equivalence to a concentration of the control IgG, IgM, or IgA antibody.
