# Supplementary material for: Longitudinal COVID-19 Surveillance and Characterization in the Workplace with Public Health and Diagnostic Endpoints
Source: mSphere. 2021 Jul 7;6(4):e00542-21. doi: 10.1128/mSphere.00542-21 (PMC8386432; doi:10.1128/mSphere.00542-21)
Supplement: TEXT S2 [file msphere.00542-21-s0002.docx]

**Longitudinal COVID-19 Surveillance and Characterization in the Workplace with Public Health and Diagnostic Endpoints**

Manjula Gunawardana,^1^ Jessica Breslin,^1†^ John M. Cortez, Jr.,^1†^ Sofia Rivera,^1†^ Simon Webster,^1†^ F. Javier Ibarrondo,^2^ Otto O. Yang,^2,3^ Richard B. Pyles,^4,5^ Christina M. Ramirez,^6^ Amy P. Adler,^7^ Peter A. Anton,^1^ and Marc M. Baum^1*^

^1^Department of Chemistry, Oak Crest Institute of Science, 128-132 W. Chestnut Ave., Monrovia, California, United States of America

^2^University of California, Los Angeles (UCLA), Division of Infectious Diseases, Department of Medicine, David Geffen School of Medicine at UCLA, Los Angeles, California, United States of America

^3^University of California, Los Angeles (UCLA), Department of Microbiology, Immunology, and Molecular Genetics, David Geffen School of Medicine at UCLA, Los Angeles, California, United States of America

^4^Department of Pediatrics, University of Texas Medical Branch, Galveston, Texas, United States of America

^5^Department of Microbiology and Immunology, University of Texas Medical Branch, Galveston, Texas, United States of America

^6^University of California, Los Angeles (UCLA), Department of Biostatistics, Fielding School of Public Health, UCLA, Los Angeles, California, United States of America

^7^Jumpstart Research Consulting, LLC, Santa Fe, New Mexico, United States of America

**Supplemental Material**

**Results**

**Sample Collection Efficiency**

Due to a shortage of appropriate swabs at the onset of the study, 5 different swab types –each tested prior to use as described in the **Methods and Materials** section– were used. **Figure S2** compares the distribution of *RP* gene transcript *Ct* values over the 3-month study as a function of swab type. Median *Ct* values consistently were below 25, suggesting efficient sample collection.
