# Supplementary material for: Longitudinal COVID-19 Surveillance and Characterization in the Workplace with Public Health and Diagnostic Endpoints
Source: mSphere. 2021 Jul 7;6(4):e00542-21. doi: 10.1128/mSphere.00542-21 (PMC8386432; doi:10.1128/mSphere.00542-21)
Supplement: TABLE S1 [file msphere.00542-21-st001.pdf]

Supplemental Table S1. Study evaluations.

	Baseline	M, W, F	Weekly	Final
Consent/assent	×			
Survey (≥ 18 years old)	×		×	×
Symptom diary	×	×		×
Nasal swab*	×	×		×
Stool swab	O		O	O
Saliva	O		O	O
Blood	O		O	O

*substitute with oral swab if unable to tolerate nasal swab.

Abbreviations: M, Monday; W, Wednesday; F, Friday; O, optional.
